# HCV NS5A Protein Containing Potential Ligands for Both Src Homology 2 and 3 Domains Enhances Autophosphorylation of Src Family Kinase Fyn in B Cells

**DOI:** 10.1371/journal.pone.0046634

**Published:** 2012-10-16

**Authors:** Kenji Nakashima, Kenji Takeuchi, Kazuyasu Chihara, Tomoko Horiguchi, Xuedong Sun, Lin Deng, Ikuo Shoji, Hak Hotta, Kiyonao Sada

**Affiliations:** 1 Division of Genome Science and Microbiology, Department of Pathological Sciences, School of Medicine, Faculty of Medical Sciences, University of Fukui, Eiheiji, Japan; 2 Organization for Life Science Advancement Programs, University of Fukui, Eiheiji, Japan; 3 Division of Microbiology, Center for Infectious Diseases, Kobe University Graduate School of Medicine, Kobe, Japan; Scripps Research Institute, United States of America

## Abstract

Hepatitis C virus (HCV) infects B lymphocytes and induces mixed cryoglobulinemia and B cell non-Hodgkin's lymphoma. The molecular mechanism for the pathogenesis of HCV infection-mediated B cell disorders remains obscure. To identify the possible role for HCV nonstructural 5A (NS5A) protein in B cells, we generated the stable B cell lines expressing Myc-His tagged NS5A. Immunoprecipitation study in the presence or absence of pervanadate (PV) implied that NS5A was tyrosine phosphorylated by pervanadate (PV) treatment of the cells. Therefore we examined pull-down assay by using glutathione S-transferase (GST)-fusion proteins of various Src homology 2 (SH2) domains, which associates with phosphotyrosine within a specific amino acid sequence. The results showed that NS5A specifically bound to SH2 domain of Fyn from PV-treated B cells in addition to Src homology 3 (SH3) domain. Substitution of Arg^176^ to Lys in the SH2 domain of Fyn abrogated this interaction. Deletion mutational analysis demonstrated that N-terminal region of NS5A was not required for the interaction with the SH2 domain of Fyn. Tyr^334^ was identified as a tyrosine phosphorylation site in NS5A. Far-western analysis revealed that SH2 domain of Fyn directly bound to NS5A. Fyn and NS5A were colocalized in the lipid raft. These results suggest that NS5A directly binds to the SH2 domain of Fyn in a tyrosine phosphorylation-dependent manner. Lastly, we showed that the expression of NS5A in B cells increased phosphorylation of activation loop tyrosine in the kinase domain of Fyn. NS5A containing ligand for both SH2 and SH3 domains enhances an aberrant autophosphorylation and kinase activity of Fyn in B cells.

## Introduction

HCV is a small enveloped positive-sense RNA virus classified within the family *Flaviviridae* [1], [2]. In addition to liver cells, HCV infects B cells, leading to mixed cryoglobulinemia and B cell non-Hodgkin's lymphoma [3]–[5]. HCV infection in B cells enhances the expression of lymphomagenesis-related genes, such as activation-induced cytidine deaminase (AID) [6], [7]. However, the molecular mechanisms of HCV infection-mediated B cell disorders remain elusive.

Non-receptor type of protein-tyrosine kinase Fyn is a member of the Src family kinases, and has regulatory roles in immune receptor signaling. Recently, Fyn has been recognized as an important mediator of mitogenic signaling and regulator of cell cycle entry, growth and proliferation. As for pathological aspects, Fyn is overexpressed in various cancers, and overexpression of Fyn in cultured cells resulted in cancer-like phenotypes [8].

The Src family kinases all share a common structure and pattern of activation. The domains of these proteins include SH2, SH3, and kinase domains followed by a short C-terminal regulatory tail. The SH2 and SH3 domains are highly conserved regions and mediate protein-protein interactions: the SH2 domain binds to phosphotyrosine residue within the specific amino acid sequence, while the SH3 domain recognizes proline rich regions. HCV NS5A was shown to interact with various SH3 domains of intracellular signaling molecules, and the kinase activity of Fyn was upregulated in liver cell lines harboring HCV replicon [9]. Binding of ligands to both the SH2 and SH3 domains disrupts autoinhibitory intramolecular interactions and leads to the opened conformation. Then autophosphorylation of the activation loop tyrosine (Tyr^420^ in Fyn) and dephosphorylation of the C-terminal tail (Tyr^531^ in Fyn) by protein-tyrosine phosphatases lead to the activation of kinase activity [10].

Previously, we reported that Syk, another non-receptor type of protein-tyrosine kinase interacts with transiently expressed NS5A in PV treated BJAB B cells [11]. This suggested that protein-tyrosine phosphorylation is required for the association of NS5A with Syk, because PV is a nonspecific inhibitor of protein-tyrosine phosphatases and treatment of cells with PV causes increase in protein-tyrosine phosphorylation in whole cells. Recently Pfannkuche *et al.* reported that NS5A binds to the SH2 domain of Src [12]. However, molecular mechanism of their interaction and effect of NS5A on the kinase activity of Src remain unclear.

In this study, we investigated the interaction between NS5A and the SH2 domain of Fyn in B cells.

## Materials and Methods

### Antibodies and cDNAs

Anti-NS5A and anti-glyceraldehyde-3-phosphate dehydrogenase (GAPDH) mAbs were purchased from Millipore (Bedford, MA, USA). Anti-Myc mAb and anti-Fyn antibody were obtained from Santa Cruz Biotechnology (Santa Cruz, CA, USA). Anti-phosphotyrosine (pTyr) (PY20) and human anti-IgM mAbs were from Zymed (South San Francisco, CA, USA). Anti-GST mAb was from Nacalai (Kyoto, Japan). Anti-phospho-Src family (Tyr416) antibody, which detects phosphorylated amount of Tyr^420^ in Fyn, was from Cell Signaling Technology (Danvers, MA, USA). The pEF1A-NS5A(Con1)-Myc-His plasmid and its deletion or substitution mutants were described previously [11]. Deletion of NS5A 127–146 (NS5A Δ127–146) was generated by the PCR-based method using four primers, 5′-TTGGTACCATGTCCGGCTCGTGGCTAAGAG-3′, 5′-GCTCTAGAGCAGCAGACGACGTCCTCA-3′, 5′-GGTTACGCGGGTGGGGGATCCCGAATTCTTCACAGAAGTG-3′, and 5′-CACTTCTGTGAAGAATTCGGGATCCCCCACCCGCGTAACC-3′, using NS5A cDNA as a template. Substitution of Tyr^129^ to Phe (Y129F) of NS5A 1–146 was generated by the site-directed mutagenesis using two primers, 5′-GGGGATTTCCACTTCGTGACGGGCA-3′ and 5′-TGCCCGTCACGAAGTGGAAATCCCC-3′, using NS5A 1–146 cDNA as a template. Substitutions of Tyr^182^ to Phe (Y182F), Tyr^321^ to Phe (Y321F), and Tyr^334^ to Phe (Y334F) of NS5A 147–447 were generated by the site-directed mutagenesis using two specific primers designed by QuikChange Primer Design Program (www.genomics.agilent.com), using NS5A 147–447 as a template. The resulted mutations were confirmed by the DNA sequencing.

### Cell culture and transfection

B-lymphoid leukemia BJAB cells were kindly provided from Dr. Satoshi Ishido (RIKEN, Yokohama, Japan) [13] and maintained as described previously [14]. For the stable transfection of BJAB cells, 6 µg of linearized pEF1A-NS5A(Con1)-Myc-His was transfected into 5×10^6^ cells/500 µl of cells by electroporation (240 V, 950 µF). Stably transfected cell lines were selected with 0.4 mg/ml of active G418 (Wako, Osaka, Japan) [15]. Cell lines were screened by level of protein expression by immunoblotting of detergent soluble lysates with anti-NS5A and anti-GAPDH mAbs as an internal control. Two positive cloned lines were selected for further analysis. For control cells, linearized empty vector was transfected by electroporation, and pooled clones resistant to 0.4 mg/ml of active G418 were utilized as control cells. COS cells were obtained from American Type Culture Collection (Manassas, VA, USA) and Ramos-T cells were kindly provided from Dr. Hamid Band (Nebraska Medical Center, NE, USA) [16]. Transient transfection of COS cells and Ramos-T cells were described previously [17]. Huh-7.5 cells were kindly provided from Dr. Charles M. Rice (The Rockefeller University, NY, USA) [18] and stably harboring an HCV replicon (pFK5B/2884 Gly) were described previously [11].

### Cell activation, immunoprecipitation and immunoblotting

BJAB cells (10^8^) were washed twice with serum free medium and treated with 100 µM PV or 10 µg/ml of anti-IgM mAb for 3 min at 37°C in the same medium. Either unstimulated or stimulated cells were washed twice with ice-cold PBS and then solubilized in the lysis buffer (1% Triton X-100, 50 mM Tris, pH7.4, 150 mM NaCl, 10 mM EDTA, 100 mM NaF, 1 mM Na_3_VO_4_, 1 mM phenylmethylsulfonyl fluoride and 2 µg/ml aprotinin) on ice. In some experiments, 0.5% Nonidet P-40 was used instead of 1% Triton. Precleared cell lysates were incubated with the indicated antibodies prebound to protein A-agarose beads (Sigma, St. Louis, MO, USA). After rotation for 90 min at 4°C, the beads were washed 4 times with the lysis buffer, and the immunoprecipitated proteins were eluted by the heat treatment for 5 min at 100°C with 2×sampling buffer. Precipitated proteins or cell lysates were separated by SDS-PAGE and transferred to polyvinylidene difluoride (PVDF) membrane (Millipore). After blocking in 5% milk in TBST (25 mM Tris, pH 8.0, 150 mM NaCl, and 0.1% Tween 20), the blots were incubated with the primary antibodies and then horseradish peroxidase-conjugated goat anti-rabbit IgG, goat anti-mouse IgG antibodies (Jackson ImmunoResearch Laboratories, West Grove, PA, USA), or horseradish peroxidase-conjugated protein G (Sigma) in TBST. To enhance the signals, Immuno-enhancer Reagent A (Wako) was utilized in the reaction with anti-pTyr (pY20) mAb. Finally, proteins were visualized by the enhanced chemiluminescence (ECL) reagent (Western Lightning, PerkinElmer Life Sciences, Boston, MA) [19]. Immunoblot quantification was performed using the program Scion Image (Scion, Frederick, MD, USA).

### Pull-down assay

The cDNA for Fyn-SH2 (Trp^149^-Ala^257^) and –SH3 (Thr^82^-Glu^148^) were amplified by PCR using paired primers 5′-GGAATTCATGGTACTTTGGAAAACTTGGC-3′ and 5′-CCGCTCGAGATCTTTAGCCAATCCAGAAGT-3′ for –SH2, 5′-GGAATTCAACAGGAGTGACACTGTTTGTG-3′ and 5′-CCGCTCGAGCTCTTCTGCCTGGATGGAGTC-3′ for –SH3, using mouse Fyn(T) cDNA (a gift from Dr. Yasuhiro Minami, Kobe University, Kobe, Japan) as a template. The cDNA for c-Abl-SH2 (Typ^146^-His^221^) and -SH3 (Leu^84^-Val^138^) were amplified by PCR using paired primers 5′-CGGAATTCCTGGTATCATGGCCCTGTATCT-3′ and 5′-ATAGTTTAGCGGCCGCTAGCTGGGTAGTGGAGTGTGGT-3′ for -SH2, 5′-CGGAATTCCCTTTTTGTGGCACTCTATGAT-3′ and 5′-TAGTTTAGCGGCCGCTGACGGGGGTGATGTAGTTGCT-3′ for -SH3, using mouse c-Abl cDNA (a gift from Dr. David Baltimore, California Institute of Technology, CA, USA) as a template. The cDNA for Cbl-b N-terminal region containing SH2 domain (Ala^2^-Pro^349^) was amplified by PCR using 5′-CGGAATTCCGCAAACTCAATGAATGGCAGA-3′ and 5′-CCGCTCGAGCTAAGGTGTAGGTTCACATAATCC-3′, using human Cbl-b cDNA (a gift form Dr. Stanley Lipkowitz, National Naval Cancer Center, MD, USA) as a template. Resulted PCR fragments were subcloned into pGEX-4T.3 (GE Healthcare, Piscataway, NJ, USA) to make domain in-frame with the downstream of GST and verified by DNA sequencing. The GST-rat Lyn-SH2 and Syk-SH2 (N+C) expression constructs were provided by Dr. Reuben P. Siraganian (National Institutes of Health, MD, USA). Preparation of GST-rat Vav1-SH2, mouse c-Abl SH3 domain-binding protein-2 (3BP2)-SH2, human phospholipase C (PLC)-γ2-SH2 (N+C), and rat Lyn-SH3 domain expression constructs were described elsewhere [17], [20], [21]. Substitution of Arg^176^ to Lys (R176K) by a point mutation of pGEX-4T.3-Fyn-SH2 was generated by the site-directed mutagenesis using two primers 5′-TCAAAGAGAGCCAAACCACCAAAGG-3′ and 5′-TAAGAAAGGTACCTCTTGGGTTTCC-3′, using Fyn-SH2 cDNA wild type as a template. The resulted point mutation was confirmed by the DNA sequencing. All these SH2 and SH3 domains were fused downstream of GST. The GST fusion proteins were affinity-purified with glutathione Sepharose 4B beads (GE Healthcare). Extraction of GST-fusion proteins from bacteria was confirmed by the SDS-PAGE and Coomassie brilliant blue staining [22].

BJAB cells (10^8^), Huh-7.5 cells stably harboring an HCV replicon (3×10^6^), COS cells (10^6^) or Ramos B cells expressing SV40 T antigen (Ramos-T cells) (10^7^) were washed twice with serum free medium and stimulated with 100 µM PV for 3 min at 37°C. Either unstimulated or stimulated cells were solubilized in the binding buffer (1% NP-40, 50 mM Tris, pH7.4, 150 mM NaCl, 10 mM EDTA, 100 mM NaF, 1 mM Na_3_VO_4_, 1 mM phenylmethylsulfonyl fluoride and 2 µg/ml aprotinin). After centrifugation, the resulted supernatants were reacted with 20 µg of GST-fusion proteins prebound to glutathione Sepharose 4B beads for 90 min at 4°C. The beads were washed 4 times with the binding buffer. Proteins interacting with GST-fusion proteins were eluted by heat treatment for 5 min at 100°C with 2×sampling buffer, separated by SDS-PAGE, and analyzed by immunoblotting.

### Far-western

Anti-NS5A immunoprecipitates from BJAB cells (3×10^7^) were separated by SDS-PAGE and transferred to PVDF membrane. After blocking, the membranes were incubated with 2.5 µg/ml of GST or GST-Fyn-SH2 for 1 h at 4°C. After extensive washing, membranes were reacted with anti-GST mAb, subsequently reacted with horseradish peroxidase conjugated goat anti-mouse IgG antibody, and then subjected to ECL detection [17].

### Subcellular fractionation

The low density detergent-insoluble fractions were prepared by sucrose density gradient centrifugation as described [23]. BJAB cells (10^8^) were solubilized in 2.5 ml of 0.05% Triton in MNEV buffer (150 mM NaCl, 25 mM Mes, pH 6.5, 5 mM EDTA, 1 mM Na_3_VO_4_, and protease inhibitors) and dounced 10 times. Homogenates were cleared of intact cells by centrifugation for 10 minutes at 200 *g*. The resultant supernatants (2.4 ml) were mixed with equal volumes of 80% sucrose in MNEV buffer (final, 40% sucrose and 0.025% Triton), overlayered by 4.8 ml 30% and 2.4 ml 5% sucrose in MNEV buffer, and then centrifuged for 20 hours at 200 000 *g* (P40ST rotor, Himac CP80WX, Hitachi, Tokyo, Japan). After sucrose density gradient centrifugation, 9 fractions were collected from the top of the gradient and analyzed by the immunoblotting.

### Statistical analysis

Quantification of Fyn was analyzed by ImageJ software. The two-tailed Student t-test was applied to evaluate the statistical significance of differences found. A *P* value of <0.05 was considered statistically significant.

### *In vitro* kinase assay

Unstimulated BJAB cells were washed twice with ice-cold PBS and then solubilized in the lysis buffer. Precleared cell lysates were incubated with anti-Fyn antibody prebound to protein A-agarose beads. After rotation for 90 min at 4°C, the beads were washed 4 times with the lysis buffer, 2 times with the kinase buffer without ATP, then incubated with 20 µl of the kinase buffer (40 mM Hepes, pH 7.5, 10 mM MgCl_2_, 2 mM MnCl_2_, 4 µM ATP, 4 µCi [γ-^32^P] ATP) and 2.5 µg of acid-treated enolase (Sigma) for 30 min at room temperature. Reaction was terminated and proteins were eluted by the heat treatment for 5 min at 100°C with 2×sampling buffer. Proteins were separated by SDS-PAGE and gel was incubated with 1N KOH for 1 h at 56°C to remove phosphoserine and most of phosphothreonine. After fixation, the gel was dried and radiolabeled proteins were visualized by autoradiography. Immunoprecipitation of Fyn was confirmed by the immunoblotting.

## Results

### HCV NS5A associates with the SH2 domain of Fyn

**Figure 1:**
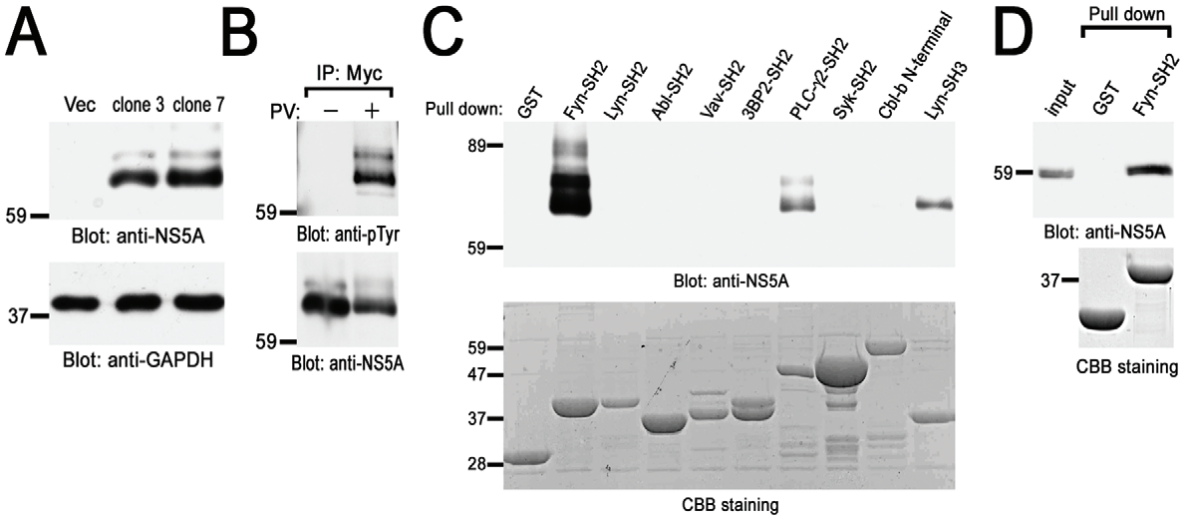
Identification of HCV NS5A-interacting proteins in B cells. (A) Generation of stable B cell lines expressing HCV NS5A. Detergent soluble cell lysates from vector cells (Vec) and Myc-His-NS5A expressing clones (clones 3 and 7) were separated by SDS-PAGE and analyzed with immunoblotting with anti-NS5A and anti-GAPDH mAbs. (B) BJAB cells expressing Myc-His-NS5A (clone 7) were treated without (−) or with (+) PV and solubilized in the lysis buffer. Cell lysates were immunoprecipitated with anti-Myc mAb and immunoprecipitated proteins were separated by SDS-PAGE and analyzed with immunoblotting with anti-pTyr (PY20) and anti-NS5A mAbs. PV-treated cells expressing Myc-His-NS5A (clone 7) (C) or Huh-7.5 cells stably harboring an HCV subgenomic replicon (D) were solubilized in the binding buffer. Precleared lysates were reacted with the indicated GST-fusion proteins and binding proteins were separated by SDS-PAGE and analyzed with immunoblotting with anti-NS5A mAb. The amount of GST-fusion proteins was confirmed by Coomassie brilliant blue (CBB) staining (C and D). Molecular sizing markers are indicated at left in kilodalton. The results were representative of three independent experiments. Similar results were obtained when another line was examined (B and C).

To identify HCV NS5A-interacting protein in B cells, we generated the stable B cell lines in which Myc-His tagged NS5A protein is constitutively expressed. Since we confirmed that the parental cells did not express NS5A, we choose the clones in which the level of NS5A expression was highest (Fig. 1A). In the following experiments, two cloned lines (clone 3 and 7) were examined, although some figures present the results from only one representative cell line. For control, vector plasmid was transfected into the same parental cells and G418-resistant clones were pooled and utilized as control cells.

Next, we performed immunoprecipitation study using anti-Myc mAb and found tyrosine phosphorylated proteins (Fig. 1B). This suggests that NS5A was tyrosine phosphorylated by PV treatment or another protein with similar size that associates with NS5A (Fig. 1B). Another experiment by affinity tag purification using Nickel column which could react with His-tag (His-Accept kit, Nacalai) also showed some tyrosine phosphorylated proteins in NS5A protein complex (data not shown). These findings suggest the possible involvement of protein-tyrosine phosphorylation associating with NS5A. Therefore, we tried to identify the protein which associates with NS5A through SH2 domain, which recognizes specific phosphotyrosine-containing amino acid sequence.

Then we carried out pull-down assay using GST-fusion proteins of various SH2 domains (Fig. 1C). Among these, the SH2 domain of Fyn dramatically bound to NS5A from PV-treated B cells. The SH2 domains of PLC-γ2 weakly bound to NS5A. The SH2 domains of Lyn, Abl, Vav, 3BP2, Syk or Cbl-b interacted with NS5A at very low level (long exposure, data not shown). GST-Lyn-SH3 was utilized as positive control because it was reported to interact with NS5A [9]. Therefore, this data demonstrated that HCV NS5A selectively binds to the SH2 domains of Fyn and PLC-γ2 in B cells. GST-human Fyn-SH2 also interacted with NS5A (Fig. S1). Moreover, the NS5A interaction with GST-Fyn-SH2 was observed even in the context of HCV RNA replication (Fig. 1D). Thus, HCV NS5A selectively associates with the SH2 domain of Fyn.

### NS5A binds to the SH2 domain of Fyn in a tyrosine phosphorylation-dependent manner

**Figure 2:**
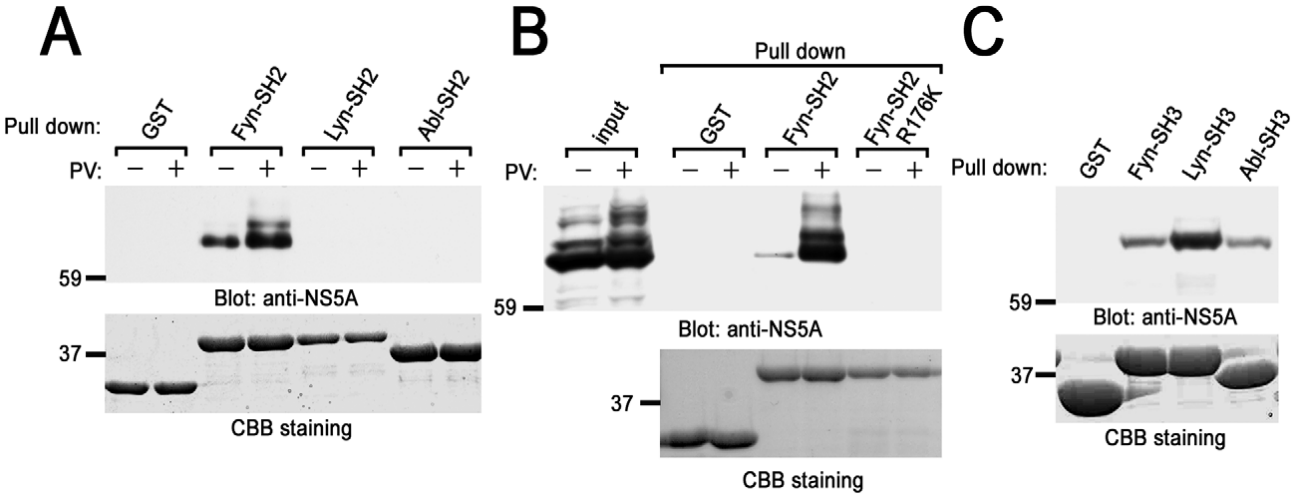
Pervanadate treatment of cells stimulates the binding of NS5A to the SH2 domain of Fyn in B cells. Either nontreated or PV-pretreated cells expressing Myc-His-NS5A (clone 7) were reacted with GST-fusion proteins of SH2 domains of various protein-tyrosine kinases (PTKs) (A), GST-Fyn-SH2 or GST-Fyn-SH2 R176K (B), or SH3 domains of various PTKs (C). Binding proteins were separated by SDS-PAGE and analyzed with immunoblotting with anti-NS5A mAb. The amount of GST-fusion proteins was confirmed by CBB staining. Molecular sizing markers are indicated at left in kilodalton. The results were representative of three independent experiments. Similar results were obtained when another line was examined.

PV treatment of cells dramatically enhances the binding of NS5A to the SH2 domain of Fyn, but not with that of Lyn or Abl (Fig. 2A). Substitution of Arg^176^ to Lys in the SH2 domain of Fyn, which caused the loss of association with phosphotyrosine residue, abrogated the binding of the SH2 domain of Fyn to NS5A (Fig. 2B). Arg^176^ is located in the consensus sequence within the SH2 domains to interact with phosphotyrosine residue. On the other hand, the SH3 domain of these kinases associated with NS5A to a comparable level (Fig. 2C). These results suggest that increase in tyrosine phosphorylation of NS5A itself, or other associating proteins, allow the interaction with the SH2 domain of Fyn.

### Central and/or C-terminal regions of NS5A binds to the SH2 domain of Fyn

**Figure 3:**
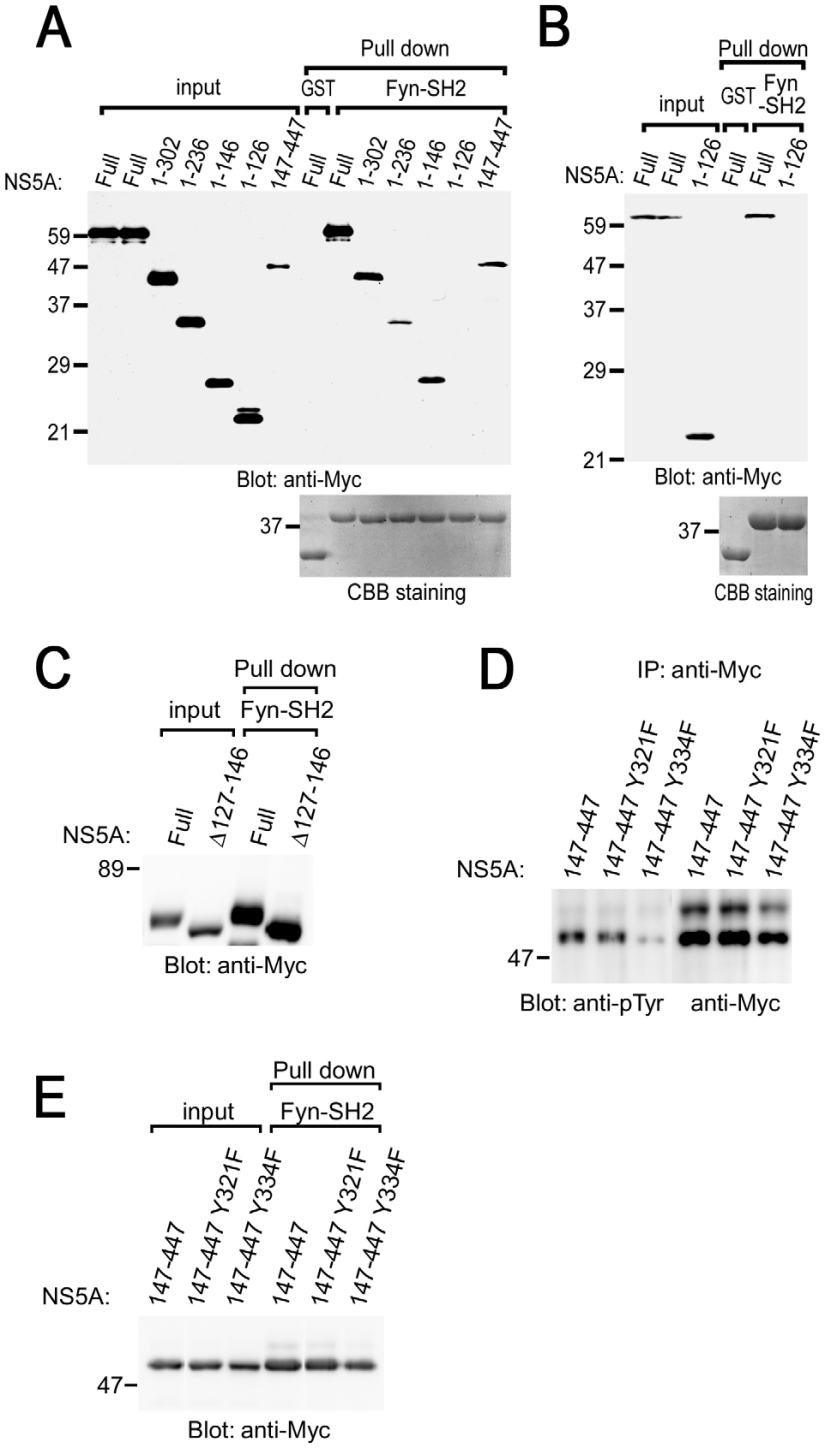
Structural analysis of the association of NS5A with the SH2 domain of Fyn in B cells. COS cells (A, C, E) or B cells (Ramos-T) (B) expressing different kinds of NS5A were stimulated with PV and subjected to pull-down assay using GST-Fyn-SH2. Binding proteins were separated by SDS-PAGE and analyzed with immunoblotting with anti-Myc mAb. The amount of GST-fusion proteins was confirmed by CBB staining. (D) COS cells expressing different kinds of NS5A mutants were stimulated with PV and subjected to immunoprecipitation. Precipitated proteins were separated by SDS-PAGE and analyzed with immunoblotting with anti-pTyr (PY20) and anti-Myc mAbs. Molecular sizing markers are indicated at left in kilodalton. The results were representative of three independent experiments.

To map the Fyn-SH2-binding region in NS5A, a series of deletion mutants were examined (Fig. 3). The results obtained reveals that N-terminal region (amino acids number 1–126) is not required for the interaction with the SH2 domain of Fyn in COS cells, although this region contains the region to interact with another kinase Syk (Fig. 3A) [11]. NS5A 127–146 and 147–447 could interact with the SH2 domain of Fyn. This demonstrates that deletion of the Fyn-binding region in the context of the full-length protein leads to loss of function. Similar results were obtained when HCV NS5A proteins were transiently expressed in Ramos B cells expressing SV40 T antigen (Ramos-T cells), and examined by pull-down assay (Fig. 3B). Deletion of 127–146 (NS5A Δ127–146) still allowed binding of NS5A to the SH2 domain of Fyn (Fig. 3C). This suggests that NS5A Δ127–146 could interact with the SH2 domain of Fyn through NS5A 147–447.

### Identification of Tyr^334^ as a tyrosine phosphorylation site in NS5A

In COS cells, full length and a series of deletion mutants of NS5A were tyrosine phosphorylated by PV treatment (Fig. S2). Because NS5A 1–126 could not bind to the SH2 domain of Fyn, we examined the possible involvement of tyrosine residue between amino acids number 127 and 146 for the binding. In this region, there is only one tyrosine residue that appears to be conserved. However, substitution of Tyr^129^ to Phe of truncated NS5A (NS5A 1–146 Y129F) still allowed tyrosine phosphorylation by PV and binding to the SH2 domain of Fyn in COS cells (Fig. S2 and S3). Thus, Tyr^129^ is not critical for the binding of NS5A to the SH2 domain of Fyn. In addition to this region (127 to 146), we examined the conserved tyrosine residues between 147 and 447 of NS5A (Tyr^182^, Tyr^321^, and Tyr^334^). Among those, substitution of Tyr^334^ to Phe (Y334F) reduced tyrosine phosphorylation of NS5A 147–447, however this mutant could interact with the SH2 domain of Fyn (Fig. 3D and E). NS5A Y182F and Y321F were tyrosine phosphorylated as NS5A 147–447 (Fig. 3D and data now shown). Therefore, these results suggest that Tyr^334^ is required for tyrosine phosphorylation of NS5A, and existence of the multiple mechanisms for the binding of NS5A with Fyn including pTyr-SH2 domain interaction. Furthermore, we could not detect the increase in tyrosine phosphorylation of NS5A by *in vitro* kinase reaction with Fyn (data not shown), suggesting that some other protein-tyrosine kinases are required for phosphorylating NS5A.

### The SH2 domain of Fyn directly binds to NS5A

**Figure 4:**
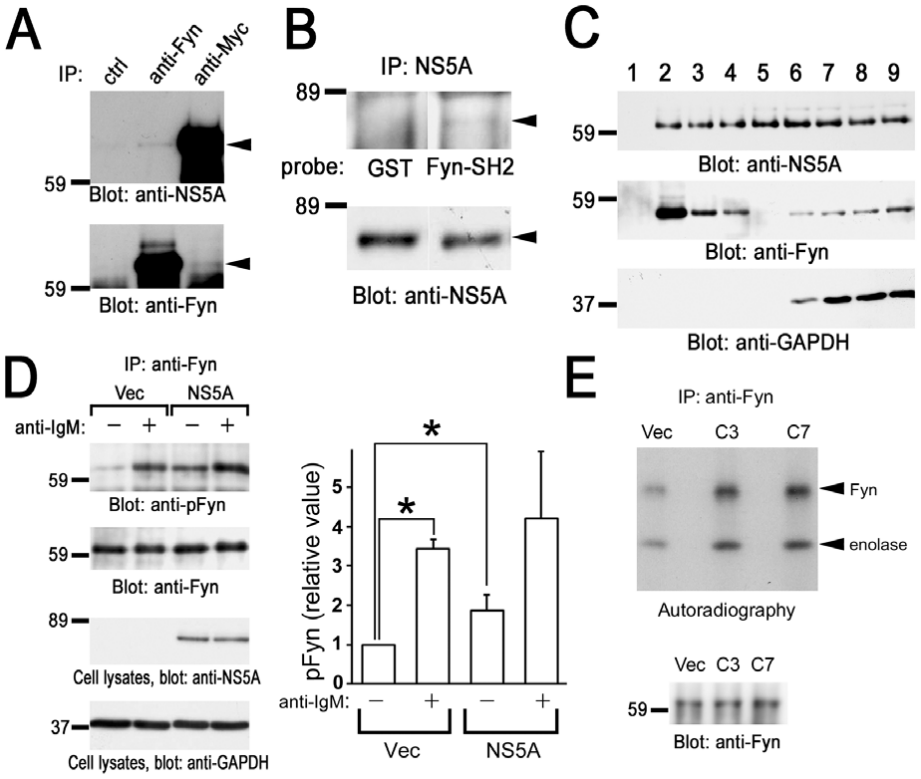
Association with NS5A increases the kinase activity of Fyn in B cells. (A) Endogenous interaction of Fyn with NS5A in BJAB cells. Cells were solubilized in the lysis buffer containing 0.5% Nonidet P-40. Detergent-soluble lysates from BJAB cells expressing Myc-His-NS5A (clone 7) were subjected to immunoprecipitation with anti-Fyn or anti-Myc antibodies. Protein interactions between NS5A and Fyn were analyzed by the immunoblotting with anti-NS5A mAb and anti-Fyn antibody, respectively. (B) Anti-Myc immunoprecipitates were separated by SDS-PAGE and subjected to far western analysis with GST or GST-Fyn-SH2 (GST-Fyn-SH2) (upper panel), and immunoblotting analysis with anti-NS5A mAb (lower panel). (C) Cell homogenates were fractionated by sucrose density gradient centrifugation. Proteins from these fractions were separated by SDS-PAGE and analyzed with immunoblotting with anti-NS5A mAb, anti-Fyn, and anti-GAPDH antibodies. (D) Control cells (Vec) and cells expressing Myc-His-NS5A (clone 7) were unstimulated (−) or stimulated (+) with anti-IgM mAb. Anti-Fyn immunoprecipitates (IP) were separated by SDS-PAGE and analyzed by immunoblotting with anti-phospho-Src family (Tyr416) antibody recognizing autophosphorylated Fyn (pFyn) and anti-Fyn antibody. Detergent-soluble lysates were separated by SDS-PAGE and analyzed by immunoblotting with anti-NS5A and anti-GAPDH mAbs. Densitometry analysis was performed on three experiments representative of Fig. 4D. Levels of pFyn were normalized to their respective total Fyn protein. The fold changes of pFyn are shown relative to unstimulated control cells. Data represent the mean ± SD of three independent experiments. *, *P*<0.05. (E) Anti-Fyn immunoprecipitates (IP) from control cells (Vec), cells expressing Myc-His-NS5A clone 3 (C3) and clone 7 (C7) were subjected to *in vitro* kinase assay using enolase as an exogenous substrate. Radioactive proteins were separated by SDS-PAGE and visualized by autoradiography. Immunoprecipitated Fyn was analyzed by immunoblotting. Molecular sizing markers are indicated at left in kilodalton. The results were representative of three independent experiments. Similar results were obtained when another line was examined.

Next we examined the mechanism of the interaction of the SH2 domain of Fyn and NS5A. Association of Fyn and NS5A in B cells were tested by the immunoprecipitation study (Fig. 4A). The result showed that NS5A was coprecipitated with anti-Fyn antibody, and vice versa. Therefore, NS5A complexes with Fyn in B cells. Far-western analysis further demonstrated that the SH2 domain of Fyn could directly bind to NS5A, suggesting that NS5A is tyrosine phosphorylated in B cells (Fig. 4B). We have shown that PV treatment of cells stimulates tyrosine phosphorylation of NS5A (Fig. 1B). These results demonstrate that NS5A could be tyrosine phosphorylated in B cells and directly associated with the SH2 domain of Fyn. In addition, we demonstrated the subcellular fractionation by sucrose density gradient centrifugation (Fig. 4C). Fractions 2–4 were regarded as low density detergent-insoluble fractions, whereas 5–9 were detergent-soluble fractions. As shown, NS5A broadly located in almost all of the fractions. In contrast, most of Fyn was located in detergent-insoluble fractions because of the lipid modification of Fyn (fractions 2–4), and some were in the detergent-soluble fractions (fractions 5–9). These results demonstrate that some of NS5A and Fyn are located in low density detergent-insoluble fractions in B cells.

### Association with NS5A increases autophosphorylation and kinase activity of Fyn

Finally, we examined the effect of the expression of NS5A on the function of Fyn tyrosine kinase (Fig. 4D). Cross-linking of B cell receptor by anti-IgM mAb resulted in the increase in phosphorylation of a tyrosine residue in the activation loop of the kinase domain of Fyn, which parallels to its kinase activity [10]. Immunoprecipitation and immunoblotting experiments demonstrated that coexpression of NS5A increases phosphorylation of activation loop tyrosine and anti-IgM stimulation enhances this phenomenon. Immunoblot quantification also indicated the significant higher phosphorylation of the activation loop of Fyn in the unstimulated state in the NS5A expressing cells. In addition, we examined the biochemical kinase activity of Fyn by *in vitro* kinase assay (Fig. 4E). Coexpression of NS5A enhanced the kinase activity of Fyn as measured by both autophosphorylation and phosphorylation of exogenous substrate (enolase). This result biochemically demonstrated that association with NS5A increases tyrosine kinase activity of Fyn to phosphorylate tyrosine residues on Fyn itself and exogenous substrate. These results suggest that association of NS5A enhances an autophosphorylation and kinase activity of Fyn in B cells.

## Discussion

We have demonstrated the possible tyrosine phosphorylation of NS5A in B cells and the interaction of NS5A with the SH2 domain of Fyn, in addition to SH3 domain. Previous reports demonstrated that cells harboring HCV replicon possesses the increased kinase activity of Fyn, which supports our conclusion of this study [9]. NS5A contains a highly conserved proline rich regions with Pro-X-X-Pro-X-Arg motif which is capable of the interaction with the SH3 domains of variety of cellular proteins, including Fyn [24]. Our finding reveals the second interaction site of Fyn to associate with NS5A. Therefore, NS5A could associate with both SH3 and SH2 domains. Through the two interactions, via SH3 and SH2 domains, it is predicted that NS5A could alter the conformation of Fyn to open active state. Physiological mechanism has generally been recognized that adaptor proteins with ligands of SH2 and SH3 domains bind to Src family kinases and positively regulates the kinase activity. Consistent with previous reports, our study demonstrated that NS5A protein containing potential ligands for both SH3 and SH2 domains increases autophosphorylation of Fyn in B cells.

Fyn has two tyrosine phosphorylation sites; one tyrosine in the activation loop is phosphorylated by autophosphorylation and the other in the C-terminal tail is phosphorylated by Csk to negatively regulate the kinase activity. In this manuscript, we examine the phosphorylation of tyrosine in the activation loop by using anti-phospho-Src family (Tyr416) antibody, which detects phosphorylated amount of a conserved tyrosine in the activation loop of Src family kinase (Fig. 4D). Therefore, we could conclude that tyrosine phosphorylation of Fyn was occurred in Tyr^420^ in the kinase domain.

The small interference RNA library screening study demonstrated that Csk is one of the protein-tyrosine kinases involved in the replication of HCV [25]. Csk is known to phosphorylate tyrosine residue in the C-terminal tail and negatively regulate Src family kinase, such as Fyn. Knock down of Fyn resulted in up-regulation of HCV replication [25]. This suggests that activation of Fyn suppresses HCV replication. In light of the aberrant increase in autophosphorylation of Fyn by NS5A coexpression, it is possible that NS5A negatively regulates HCV replication with activating Fyn kinase assumedly for persistent infection.

v-Src is the first discovered oncogene, and Fyn is a member of cellular Src family kinases and is also associated with cancer. Overexpression of Fyn in NIH3T3 fibroblast cells exhibited a cancer-like phonotype with increased anchorage-independent growth and prominent morphologic changes. Other studies have revealed that overexpression of Fyn results in promotion of the anti-apoptotic activity of Akt and dysregulation of anchorage-dependent cell growth. In this study, expression of NS5A enhanced autophosphorylation of Fyn in B cells, suggesting that HCV-mediated activation of Fyn can promote aberrant growth and anti-apoptotic status leading to B lymphomagenesis [8].

Adaptor proteins have also been recognized candidates to promote oncogenes. For example, v-Crk (CT10 regulator of tyrosine kinase)/Crk-I are adaptor proteins composed of SH2 and SH3 domains but lack negative regulatory region (phosphotyrosine and C-terminal SH3 domain). Those adaptors function as constitutively activated ones, leading to tumorgenesis. Like that, NS5A presumably works constitutive activated adaptor for Fyn kinase [26].

In conclusion, present study demonstrated that NS5A binds to the SH2 domain of Fyn in tyrosine phosphorylation-dependent manner and that NS5A containing ligand for both SH2 and SH3 domains produces an aberrant increase in autophosphorylation and kinase activity of Fyn. Further studies are needed to clarify which tyrosine residues in NS5A are phosphorylated and bind to SH2 domain of Fyn. These data, however, may contribute to our understanding of the mechanisms that HCV infection causes B lymphomagenesis.

## Supporting Information

Figure S1 **GST-human Fyn-SH2 could react with NS5A.** The cDNA for human Fyn-SH2 (Trp^149^-Arg^268^) were amplified by PCR using paired primers 5′-GGAATTCATGGTACTTTGGAAAACTTGGC-3′ and 5′-GATCAACTGCAGGGATTCTCG -3′, using cDNA from total RNA of BJAB cells as a template. Resulted PCR fragment was subcloned into the pGEX-4T.3 (GE Healthcare) to make domain in-frame with the downstream of GST and verified by DNA sequencing. PV-treated cells expressing Myc-His-NS5A (clone 7) were solubilized in the lysis buffer. Precleared lysates were reacted with GST or GST-human Fyn-SH2 and binding proteins were separated by SDS-PAGE and analyzed with immunoblotting with anti-NS5A mAb. The amount of GST-fusion proteins was confirmed by CBB staining. Molecular sizing markers are indicated at left in kilodalton. The results are representative of two independent experiments.
(TIF)

Figure S2 **Tyrosine phosphorylation of NS5A and its mutants in COS cells.** Full length and a series of deletion mutants of NS5A were transiently expressed in COS cells. Cells were unstimulated (−) or stimulated (+) with PV and solubilized in the lysis buffer. Cell lysates were immunoprecipitated with anti-Myc mAb and immunoprecipitated proteins were separated by SDS-PAGE and analyzed with immunoblotting with anti-pTyr (PY20) and anti-Myc mAbs. Molecular sizing markers are indicated at left in kilodalton. The results were representative of three independent experiments.
(TIF)

Figure S3 **Tyr^129^ is not critical for the binding of NS5A to the SH2 domain of Fyn.** Indicated mutant forms of NS5A were transiently expressed in COS cells. Cells were unstimulated (−) or stimulated (+) with PV. Cells were solubilized in the binding buffer and precleared lysates were reacted with GST-Fyn-SH2. Detergent-soluble lysates and binding proteins were separated by SDS-PAGE and analyzed with immunoblotting with anti-Myc mAb. Molecular sizing markers are indicated at left in kilodalton. The results were representative of three independent experiments.
(TIF)
