# Supplementary figures and images for: HCV NS5A Protein Containing Potential Ligands for Both Src Homology 2 and 3 Domains Enhances Autophosphorylation of Src Family Kinase Fyn in B Cells
Source: PLoS One. 2012 Oct 16;7(10):e46634. doi: 10.1371/journal.pone.0046634 (PMC3473057; doi:10.1371/journal.pone.0046634)

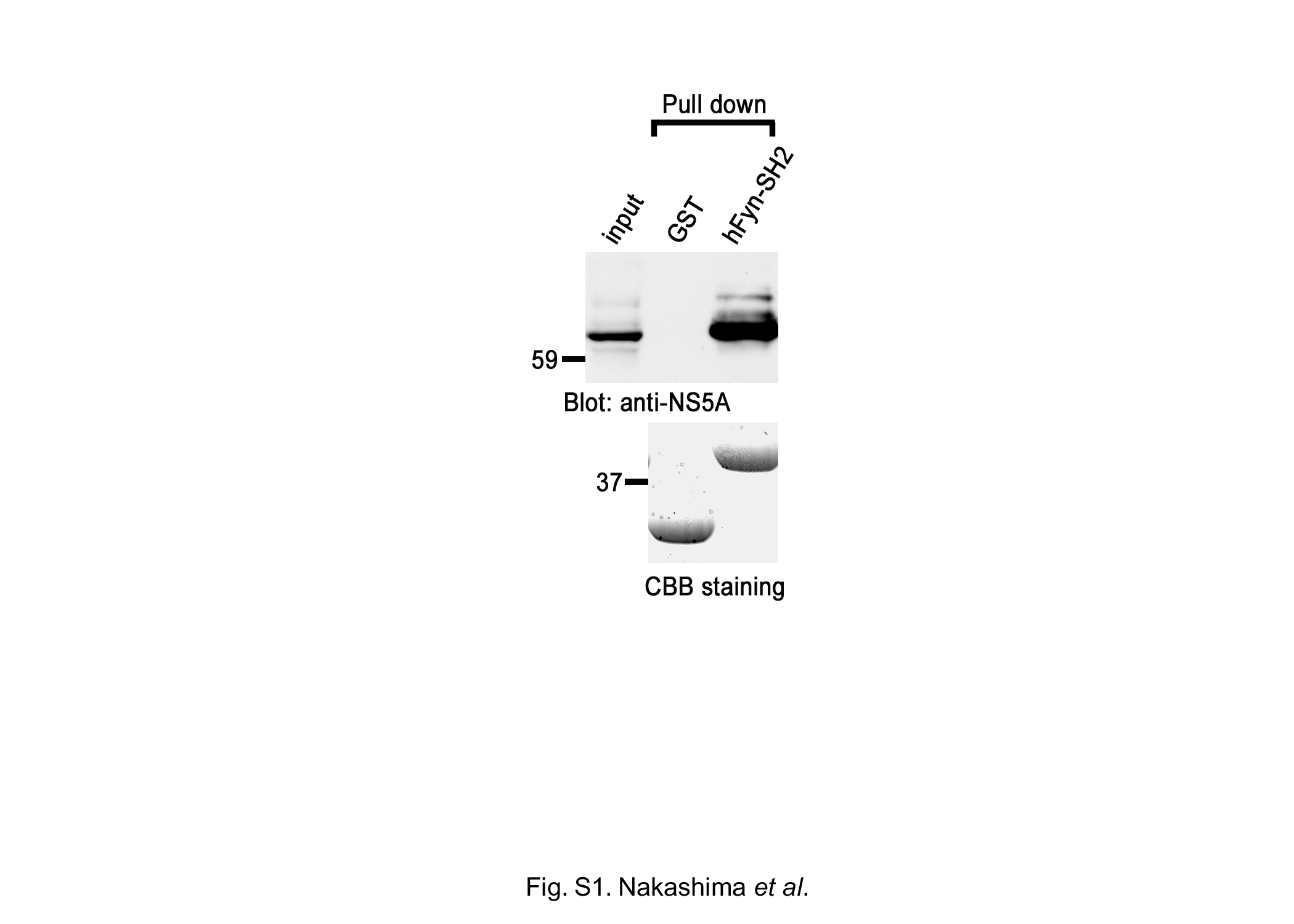

Supplement: Figure S1 — GST-human Fyn-SH2 could react with NS5A. The cDNA for human Fyn-SH2 (Trp149-Arg268) were amplified by PCR using paired primers 5′-GGAATTCATGGTACTTTGGAAAACTTGGC-3′ and 5′-GATCAACTGCAGGGATTCTCG -3′, using cDNA from total RNA of BJAB cells as a template. Resulted PCR fragment was subcloned into the pGEX-4T.3 (GE Healthcare) to make domain in-frame with the downstream of GST and verified by DNA sequencing. PV-treated cells expressing Myc-His-NS5A (clone 7) were solubilized in the lysis buffer. Precleared lysates were reacted with GST or GST-human Fyn-SH2 and binding proteins were separated by SDS-PAGE and analyzed with immunoblotting with anti-NS5A mAb. The amount of GST-fusion proteins was confirmed by CBB staining. Molecular sizing markers are indicated at left in kilodalton. The results are representative of two independent experiments. (TIF) [file pone.0046634.s001.tif]

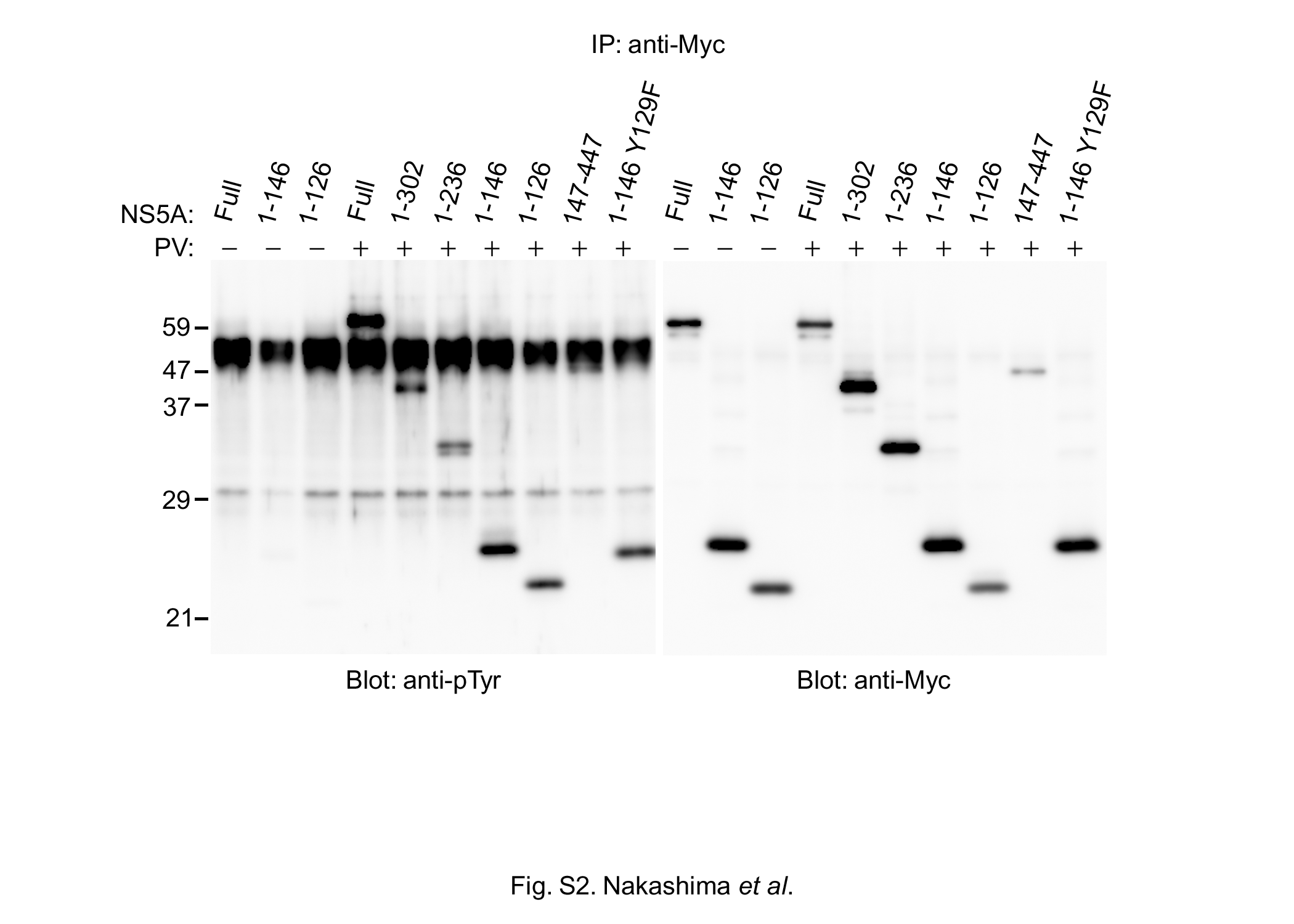

Supplement: Figure S2 — Tyrosine phosphorylation of NS5A and its mutants in COS cells. Full length and a series of deletion mutants of NS5A were transiently expressed in COS cells. Cells were unstimulated (−) or stimulated (+) with PV and solubilized in the lysis buffer. Cell lysates were immunoprecipitated with anti-Myc mAb and immunoprecipitated proteins were separated by SDS-PAGE and analyzed with immunoblotting with anti-pTyr (PY20) and anti-Myc mAbs. Molecular sizing markers are indicated at left in kilodalton. The results were representative of three independent experiments. (TIF) [file pone.0046634.s002.tif]

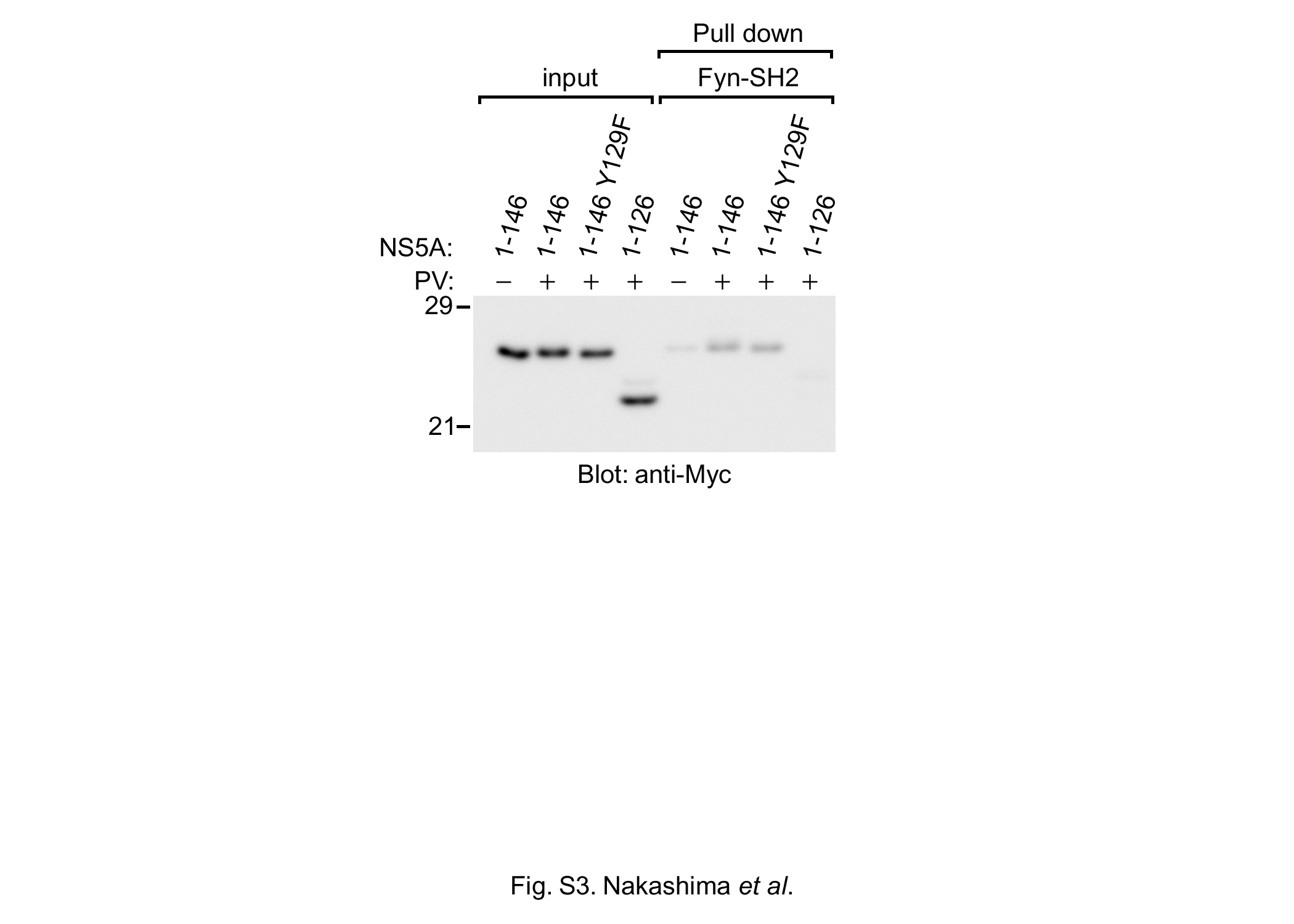

Supplement: Figure S3 — Tyr129 is not critical for the binding of NS5A to the SH2 domain of Fyn. Indicated mutant forms of NS5A were transiently expressed in COS cells. Cells were unstimulated (−) or stimulated (+) with PV. Cells were solubilized in the binding buffer and precleared lysates were reacted with GST-Fyn-SH2. Detergent-soluble lysates and binding proteins were separated by SDS-PAGE and analyzed with immunoblotting with anti-Myc mAb. Molecular sizing markers are indicated at left in kilodalton. The results were representative of three independent experiments. (TIF) [file pone.0046634.s003.tif]
